# Use of Methylphenidate to Improve Cognition in Autism Spectrum Disorder (ASD)

**DOI:** 10.1192/j.eurpsy.2023.476

**Published:** 2023-07-19

**Authors:** S. Ashraf, A. Bachu, S. Srinivas, W. Tankersley, K. Shah

**Affiliations:** ^1^Northpointe Psychiatry, Lewisville, TX; ^2^Psychiatry, UAMS-Baptist Health, North Little Rock, AR, United States; ^3^A.J.Institute of Medical Sciences and Research Center, Mangaluru, Karnataka, India; ^4^Psychiatry, Children’s Recovery Center, ODMHSAS, Norman, OK; ^5^Psychiatry, Wake Forest University, Winston-Salem, NC, United States

## Abstract

**Introduction:**

Methylphenidate (MPH) is used to treat attention deficit hyperactivity disorder (ADHD) and has shown unique benefits in children and adolescents with autism spectrum disorder (ASD). In addition to improving attention, hyperactivity, and repetitive movement, it also improves cognition in ASD. Our focus is to explore the understudied benefits and safety of methylphenidate use for cognition in ASD children.

**Objectives:**

1) To study the response and benefit of methylphenidate in autistic kids to improve cognition and life function.

2) To assess the safety profile and risks of using methylphenidate at different doses in children with autism as a guiding tool for prescribing physicians.

**Methods:**

A literature search was conducted using relevant medical subject headings (MeSH) terms in PubMed, Medline, and PubMed Central. We identified all published articles from inception until September 30, 2022. Initial search results found 41 studies, of which 15 were excluded as they did not meet inclusion and exclusion criteria. After a thorough full-text review, we included 5 studies in our final qualitative synthesis review.

**Results:**

Methylphenidate was found to have the unique benefit of increasing cognitive processing speed, thus improving everyday life function in ASD and ADHD children compared with only ADHD, with a clinical significance of p<0.001. Stimulants may improve the processing of social situations and interactions or social functions due to this unique response (Peled, J et al. *Nordic journal of psychiatry* 2020; *74*(3), 163–167) A clinically significant performance gain on cognitive tasks was identified with a linear dose-response at three different doses (10 mg, 15 mg, 20 mg) of methylphenidate. ASD children with ADHD made significantly fewer omission errors, [F(3,69) = 7.21, p < 0.001], and commission errors [F(3,69) = 4.64, p = 0.005] on MPH, compared to placebo. They also showed more response at higher MPH doses, [F(3,69) = 10.45, p < 0.001]. Thus, the children were faster, more accurate, and had fewer errors at higher MPH doses (Pearson, D. A. et al. *Journal of child and adolescent psychopharmacology* 2020; *30*(7), 414–426). There were no serious side effects or suicidality reported for low dose and a medium dose of MPH in ASD children assessed with Response Impressions and Side Effects Checklist-Kids (RISC-K), A 38-item parent rating scale (Kim, S. J. et al. Journal of autism and developmental disorders 2017; 47(8), 2307–2313).

**Conclusions:**

The findings support the positive response of methylphenidate on cognitive function in ASD children. No serious adverse effects or suicidality were noted. Multi-center well-designed studies are recommended to determine further efficacy and safety of MPH in ASD children for cognition.

**Disclosure of Interest:**

None Declared

